# Challenges in Lung Isolation in a Tracheostomized Patient: A Case Report

**DOI:** 10.7759/cureus.69668

**Published:** 2024-09-18

**Authors:** Luisa Fernandes, Ana Catarina Freitas, Mariana Azevedo

**Affiliations:** 1 Anaesthesiology, Hospital Central do Funchal, Funchal, PRT

**Keywords:** bronchial blocker, double lumen tube, lung isolation techniques, one-lung isolation, post tracheostomy, tracheostomized patients

## Abstract

Tracheostomized patients who require one-lung ventilation represent a unique challenge for the anaesthesiologist: on the one hand, the need to protect the airway safely and, on the other, provide optimal lung isolation for surgery.

A 65-year-old tracheostomized patient scheduled for an esophagectomy requiring one-lung ventilation raised a challenge in lung isolation using a bronchial blocker. The authors describe the strategies applied to overcome the obstacle and review lung isolation techniques in this type of patient.

## Introduction

Lung isolation techniques for providing one-lung ventilation (OLV) are typically necessary to enhance surgical exposure in various types of surgery, including thoracic surgeries [1]. The most common methods for achieving OLV include using a double-lumen tube (DLT) or a bronchial blocker (BB) via the oral route [1]. However, in patients with a permanent tracheostomy, such as those who have undergone a total laryngectomy, traditional OLV airway management techniques are limited [2, 3]. The shortened trachea and an unusual angle at the tracheostomy site can render intubation with a DLT impossible or unsafe [3, 4]. On the other hand, a BB is suitable for many situations but also has certain disadvantages, such as difficulties in positioning, frequent dislodgment, limited suction, and slow lung collapse [5, 6]. Anesthesiologists face a unique challenge when managing tracheostomized patients requiring OLV for surgery, as they must balance the need for safe airway protection with the need for optimal lung isolation [7].

We describe the case of a tracheostomized patient scheduled for an esophagectomy requiring OLV and the difficulties encountered in the lung isolation technique. A review of the possible techniques for lung isolation in this type of patient is presented. We believe this can aid in safely managing tracheostomized patients undergoing surgical procedures that require OLV.

## Case presentation

**Figure 1:**
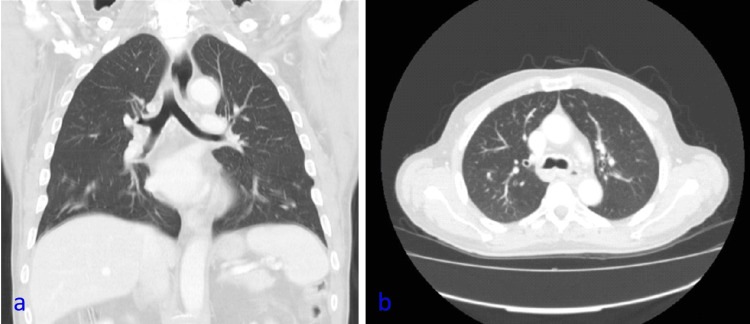
Chest CT scan a - coronal plane; b - axial plane: showing thickening of the esophageal wall and heterogeneity at the level of the carina

A 65-year-old man was scheduled for laparoscopic and thoracoscopic Ivor Lewis esophagectomy, following the diagnosis of squamous cell carcinoma of the esophagus and after neoadjuvant chemotherapy and radiotherapy, completed one month earlier. He was categorized as an American Society of Anesthesiologists (ASA) Physical Status Classification III, given the history of carcinoma of the supraglottic larynx, having undergone chemotherapy and radiotherapy 15 years prior, with a tracheostomy performed at that time. In addition, he had high blood pressure, dyslipidemia, and a previous history of smoking. His Assess Respiratory Risk in Surgical Patients in Catalonia (ARISCAT) score was 50 points, with a high risk (42.1%) of in-hospital post-op pulmonary complications. The computed tomography (CT) of the chest showed significant thickening of the esophageal wall (maximum thickness of 10 mm) and heterogeneity at the level of the carina, which extended over at least 5 cm [Figure 1]. The tracheal width immediately distal to the tracheostomy cannula was 10.02 mm and its anteroposterior diameter was 16.14 mm, while the width of the left main bronchus at the level of the carina was 10.62 mm and its anteroposterior diameter was 8.47 mm. At the same level, the right main bronchus measured 10.65 mm in width and 11.21 mm in anteroposterior diameter.

Preoperative bronchoscopy revealed an enlarged carina, with hyperemic and irregular mucosa, free left main bronchus with slightly hyperemic and irregular mucosa in the proximal posterior aspect, with convergence of endobronchial mucosal folds.

Preoperative laboratory tests didn't show any significant alterations. Assessment of functional capacity revealed a good functional capacity with metabolic equivalents (METs) of 7-9. The electrocardiogram revealed a right bundle branch block but with normal transthoracic echocardiography.

A total intravenous general anesthesia was used for the procedure, with the placement of an inner diameter 8 mm reinforced endotracheal tube (ETT) through the tracheostoma without difficulty. As the first approach to lung isolation in the thoracoscopic surgical phase, we chose to use the 9.0FR UniblockerTM (Teleflex Medical Europe Ltd) bronchial blocker, the only one available in our surgical center, guided by flexible fiberscope (Karl Storz - outer diameter at distal end 5.2 mm). However, despite the easy progression of the bronchial blocker, right lung isolation was not achieved, nor was it possible to progress the fiberscope for visualization, due to intraluminal space conflict in the ETT.

The following attempts included the replacement of the reinforced ETT with a simple polyvinyl ETT of an inner diameter of 8 mm (maximum diameter available) and another attempt to place the bronchial blocker and fiberscope, but it was unsuccessful due to the difficulty in working with both devices inside the ETT. The “blind” technique, likewise, did not obtain lung isolation confirmed by the auscultatory method.

It was finally decided to selectively intubate the left main bronchus guided by fibroscopy, to obtain right lung isolation. During the procedure, some adjustments to the positioning of the ETT were necessary due to the displacement of the tube’s cuff, causing unsatisfactory lung isolation. Added to this, lung deflation was not possible, making surgical conditions not ideal. Even so, the surgery was completed and the ETT was replaced with a non-fenestrated tracheostomy cannula (Portex®- inner diameter 10 mm) and the patient was transferred to the intensive care unit, under sedation.

## Discussion

This case illustrates the difficulty that is encountered sometimes while performing lung isolation techniques in tracheostomized patients. In addition to this challenge, the patient had previously undergone local radiotherapy, probably causing regional tissue fibrosis. The recognition of a potentially difficult airway, such as that in a patient who underwent cervical radiotherapy or head and neck surgery, before the use of lung isolation techniques, is essential. Additionally, this type of patient may present with distorted anatomy at the level of the carina or distally, caused, for example, by an extraluminal tumor, close to the tracheobronchial bifurcation, which may complicate lung isolation techniques [2]. 

In this case, we encountered some difficulties in achieving lung isolation using a BB with one cuff, which led us to place a single-lumen endotracheal tube into the left main bronchus. This technique may not be ideal in cases requiring left lung isolation due to occlusion of the orifice of the right upper lobe and subsequent hypoxemia, risk of mucosal ischemic injury due to cuff overinflation, slow and poor deflation of the lung, risk of re-inflation with multiple manipulations of the tube in the tracheobronchial tree and possibility of lung contamination [1, 3, 5]. Another alternative to avoid the conflict of inner ETT space encounter would have been using from the beginning a cuffed tracheostomy cannula of larger size, instead of the endotracheal tube, and attempting to pass a bronchial blocker with guidance of the fiberscope through the cannula. In this matter, Garg et al. reported successful airway management for OLV in a patient with a total laryngectomy using an endobronchial blocker inserted through an adult silicone hyperflex adjustable flange tracheostomy tube (SHATT) [3]. Its long and flexible, wire-reinforced silicone shaft is designed to accommodate difficult airway anatomy, providing a secure airway, intended for improved kink and crush resistance, and benefit in preventing airway morbidity in patients who had radiation exposure to cancer management [3].

In patients with a tracheostomy, OLV is typically managed using either a BB or a DLT [2]. The altered anatomy increases the likelihood of DLT and BB malposition, making it more challenging to maintain proper lung separation and isolation [2, 3]. Factors such as patient comorbidities and anatomy, the expected difficulty of placement, the potential need for intervention on the operative lung, and the preferences of the surgeon and anesthesiologist often influence the decision to use one or another device [8].

BB can be inserted through the existing tracheostomy tube or through an endotracheal tube placed into the tracheostomy stoma or the oral route. DLT can be inserted through the tracheostomy stoma if it is sufficiently wide. Alternatively, if the larynx is intact, the tracheostomy tube can be removed, and a DLT can be placed via the oral route [5]. 

The advantages of using a BB include its ability to be inserted through an existing ETT or tracheostomy, the option to isolate a specific lobe, avoiding the need for ETT exchange at the end of the procedure, and minimizing airway trauma [6, 8]. However, challenges may occur due to the anatomy of the right bronchus, along with the longer placement time, the need for more frequent repositioning, and the inability to provide continuous positive airway pressure (CPAP), apneic oxygen insufflation, suctioning, or bronchoscopy to the operative lung [1, 5, 6, 8]. Numerous types of BB have been described in tracheostomized patients, including Univent tubes, Arndt endobronchial blocker system, as well as the Cohen flextip blocker, Uniblocker, EZ-blocker or Coopdech blocker [9-12]. The use of Fogarty embolectomy catheters and Foley catheters as endobronchial blockers has also been reported [13]. A fiber optically directable wire-guided endobronchial blocker (WEB, Cook Critical Care) in association with a tracheostomy tube has been described by Mathews et al. In this approach, the distal end of the BB could be ‘steered’ to the right or left by rotating the proximal shaft clockwise or anticlockwise, respectively [14].

DLTs are often preferred for their ease of placement, minimal need for repositioning, and ability to provide CPAP or suction to the operative lung. However, their larger size can increase the risk of sore throat, hoarseness, and airway injuries [6, 8]. Additionally, DLTs may be unsuitable for patients with a shortened upper airway, a more constricted stoma, or those with a restricted angle of entry into the trachea [8]. Their use involves removing the tracheostomy tube, which carries the risk of creating a false lumen or causing airway failure, especially after a recent tracheostomy [9].

Indeed, it is important to consider whether it is a recent tracheostoma (a few days old, particularly the first seven days, in which the airway may be lost immediately or decannulation may occur) or long-term [9].

Campos et al. [9] suggested an algorithm to approach the lung isolation technique in tracheostomized patients, based on a study of 70 cases. In patients with a tracheostomy performed less than seven days prior, the low-pressure cuffed tracheostomy tube should be left in situ and a BB should be introduced through the tracheostomy tube. For patients with a tracheostomy performed more than seven days prior, the technique of choice is an intraluminal placement of a BB through a single-lumen ETT and the rescue technique is the advancement of a single-lumen endotracheal tube through the tracheostomy to the main bronchus or placing a left-sided DLT.

A special DLT device, available in Europe, has been specifically designed for tracheostomized patients, that is shorter and curved between the intratracheal and extratracheal parts (Rüsch Tracheopart®, Teleflex Medical Europe Ltd) [15]. These may be included in the previous algorithm described.

Considering the previously reported cases [5, 9, 11], the use of a BB seems to be the preferred method to OLV in tracheostomized patients, given its advantage of easier and safer placement in abnormal airways, especially if the tracheostoma is recent. In a recent six-year retrospective study, the use of a BB alongside a tracheostomy tube was found to be safe and practical for most tracheotomized patients, regardless of the tracheostomy's age or type. [16]. 

## Conclusions

Tracheostomized patients can be considered at risk of having a difficult airway approach during OLV and lung isolation. The authors have described a case that was managed by selectively intubating the left main bronchus. However, despite their disadvantages, BBs and DLTs are the most commonly used devices in this situation.
